# Automatic Detection of Regions in Spinach Canopies Responding to Soil Moisture Deficit Using Combined Visible and Thermal Imagery

**DOI:** 10.1371/journal.pone.0097612

**Published:** 2014-06-03

**Authors:** Shan-e-Ahmed Raza, Hazel K. Smith, Graham J. J. Clarkson, Gail Taylor, Andrew J. Thompson, John Clarkson, Nasir M. Rajpoot

**Affiliations:** 1 Department of Computer Science, University of Warwick, Coventry, United Kingdom; 2 Centre for Biological Sciences, Life Sciences, University of Southampton, Southampton, United Kingdom; 3 Vitacress Salads Ltd., Lower Link Farm, St Mary Bourne, Andover, United Kingdom; 4 Soil and Agri-Food Institute, School of Applied Sciences, Cranfield University, Bedford, United Kingdom; 5 School of Life Sciences, University of Warwick, Wellsbourne, United Kingdom; 6 Department of Computer Science and Engineering, Qatar University, Doha, Qatar; Centrum Wiskunde & Informatica (CWI) & Netherlands Institute for Systems Biology, Netherlands

## Abstract

Thermal imaging has been used in the past for remote detection of regions of canopy showing symptoms of stress, including water deficit stress. Stress indices derived from thermal images have been used as an indicator of canopy water status, but these depend on the choice of reference surfaces and environmental conditions and can be confounded by variations in complex canopy structure. Therefore, in this work, instead of using stress indices, information from thermal and visible light imagery was combined along with machine learning techniques to identify regions of canopy showing a response to soil water deficit. Thermal and visible light images of a spinach canopy with different levels of soil moisture were captured. Statistical measurements from these images were extracted and used to classify between canopies growing in well-watered soil or under soil moisture deficit using Support Vector Machines (SVM) and Gaussian Processes Classifier (GPC) and a combination of both the classifiers. The classification results show a high correlation with soil moisture. We demonstrate that regions of a spinach crop responding to soil water deficit can be identified by using machine learning techniques with a high accuracy of 97%. This method could, in principle, be applied to any crop at a range of scales.

## Introduction

Infrared thermometers have been used in the past by researchers to determine temperature differences in both individual plants and their canopies for irrigation scheduling purposes. The development of thermal imagers has extended the opportunities for analysis of thermal properties of plants and canopies [1]. The non-contact, non-destructive nature and repeatability of measurements makes thermal imaging useful in agriculture, the food industry and forestry [2], [3]. Imaging has been used as a tool in plants for predicting crop water stress, early disease detection, predicting fruit yield, bruise detection and detection of foreign bodies in food material. Under soil water deficits beyond a critical threshold, plants tend to close their stomata, and the rate of transpiration is reduced. This reduction in transpiration leads to an associated increase in leaf temperature. It also widens the range of temperature variation within the canopy which can be detected using infrared thermometry or by the use of thermal imagers [4]. There has been a lot of work focused on water stress analysis of plants using thermal imaging; however few researchers have exploited the information from the visible light images for analysis. Most of the work conducted uses stress indices [5], [6] and researchers have conducted various experiments to investigate the relationship between different stress indices and temperature values determined by thermal imaging [7], [8]. The use of thermal imaging as an indicator of plant stress has also been tested in a number of environmental conditions and the conditions best suited to its successful application have been explored. Leaf energy balance equation was formulated to estimate stomatal conductance [9], but the proposed energy balance equation was dependent on a range of environmental factors and plant variables such as emissivity of the leaf surface, air density and specific heat capacity. The complexity, and associated difficulty of measuring these variables accurately, made it difficult to obtain accurate estimates of stomatal conductance from leaf temperature. Consequently, leaf energy balance equation was rearranged to derive thermal indices based on ‘wet’ and ‘dry’ reference surfaces [10], [11], using the ‘Crop Water Stress Index’ (CWSI) [5], [6], thus making stomatal conductance more straightforward to calculate from leaf temperatures. There is a debate within the scientific community as to the ideal choice of reference surfaces and much work has been undertaken to find the best choice for reference surfaces and in what conditions they must be used [12].

The robustness, sensitivity and limitations of thermal imaging for detecting changes in stomatal conductance and leaf water status in plants has been analysed by researchers in various conditions [13]. The temperature of surfaces within the canopy is highly dependent on whether they are shaded or in direct sunlight; this variation has been investigated and various options have been suggested to minimise the effect. It was suggested that the average temperature of the canopy was more useful to reduce the effect of leaf angles and other environmental factors when compared to individual leaf temperatures [14]. Researchers have also compared various techniques for image acquisition and have performed experiments to investigate the potential of infrared thermography for irrigation scheduling and to evaluate the consistency and repeatability of measurements under a range of environmental conditions [15]. It was suggested to exclude pixels which are outside the wet-dry threshold range to allow for semi-automated analysis of a large area of canopy. In addition, the authors proposed using thermal data from shaded leaves for improved data consistency, since there is less variability in temperature within an image, and smaller errors resulting from differences in radiation absorbed by reference and transpiring shaded leaves. Variation coefficients of stress indices were found to be of considerable importance and discriminatory powers of the techniques for estimates of stomatal conductance were found to be limited. In a later study, it was proposed that sunlit leaves show a wider range of temperatures because, although natural leaf orientation has little effect on the energy balance of shaded leaves, there is a large effect on exposed leaves [16]. Based on these observations, the information from temperature distribution can be combined with the leaf orientation for thermal analysis in high resolution images.

Combining information from thermal and visible light images has the potential to provide a better estimate of stress indices and to identify regions in the canopy responding to soil water deficit. The use of thermal and visible imaging has been studied to maintain mild to moderate water stress levels in grapevine [17]. To estimate the canopy temperature, different sections of the canopy were used, including: the whole canopy, all of the sunlit canopy, the centre of the canopy and only sunlit leaves from the centre of the canopy. The best correlation between CWSI and stomatal conductance was calculated from the centre of the canopy measurements (or its sunlit fraction). The authors observed that CWSI computed with wet and dry references was the most robust index and suggested that the fusion of thermal and visible imaging can not only improve the accuracy of remote CWSI determination but also provide precise data on water status and stomatal conductance of grapevine.

Partly automated methods have also been used in the past to study plant stress indices [18]. The authors exploited colour information from visible light images to identify leaf area, as well as sunlit and shaded parts of the canopy. As a pre-processing step, images of constant temperature background were subtracted from the actual image to correct for relative errors in calibration of the camera caused by internal warming. Ground Control Points (GCPs) were manually selected to overlay the thermal image on the visible light image. Different regions in the visible light images were classified, using a supervised classification method, into pixels which represent leaves, other parts of the plant and background. Statistical parameters and stress indices were calculated based on temperature values from the corresponding classified regions of the plant. The results showed that temperature distribution can be used as an indicator of stomatal conductance and plant stress. More recently, researchers have used automated methods to estimate water status using aerial thermal images of palm tree canopies [19]. The authors used watershed segmentation of thermal images to detect the palm trees, and found the detected temperature to be a good indicator of the tree's water status.

Here, we aim to use combined information from thermal and visible light images of a spinach canopy to classify well-watered and water deficient plants. We present a new technique to enhance the ‘discriminatory power’ of thermal imaging to identify parts of the canopy which have reduced their transpiration rates in response to soil moisture deficit. Instead of using stress indices to identify stress regions, we combine information from visible light and thermal images and use machine learning techniques to classify between canopies growing in well-watered soil or under soil moisture deficit. Furthermore, we have acquired information about the light intensity and green-ness of the plant from the visible light images. These data are subsequently used, along with statistical information from thermal images, to classify between crop irrigation treatments using 1. Support Vector Machines (SVM), 2. Gaussian Processes Classifier (GPC) and 3. a combination of both classifiers. All three classifiers show promising results with the set of features extracted using combined information from thermal and visible light images.

## Materials and Methods

### Image Acquisition

**Figure 1:**
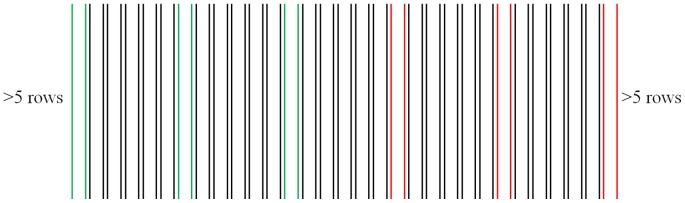
Sampling layout for the collection of thermal images and soil moisture measurements, 2010. The rows represent beds of Spinach (cv. Racoon), with those marked in green showing irrigated sample rows and the red indicating non-irrigated sample rows. Point measurements were made every 20 m for the full length of each bed (n = 54 for each treatment).

Spinach (cv. Racoon) was drilled on 11 March 2010 at Mullens Farm, Wiltshire and was maintained with commercial practice. Permission for this study was given by the farm manager (Graham Clarkson) who is also a contributing author to this manuscript. Measurements were taken on 27 April of two treatment areas in bright and clear conditions; well-watered and water-deficient. The former treatment had been irrigated during the preceding week, while the latter had not, and were both harvested the following week for market. Both treatment areas were crops of spinach of the same age and variety and both had reached full canopy cover. Sampling consisted of taking a single image and soil moisture measurement at 20 m intervals for the length of each row. Three rows were sampled per treatment, with five rows separating the sampled rows (Figure 1). Soil moisture measurements were made using a Delta-T ML2x Thetaprobe connected to a HH2 moisture meter (Delta-T Devices, Cambridge, UK), with the probe position being in the centre of the bed at a depth of approximately 7 cm. The infra-red thermal images were taken using a TH9100WR thermal camera (NEC, Metrum) from a fixed distance of approximately 1 m above the crop. The camera operated in the region of 8–14 µm with 0.1°C thermal resolution and a spatial resolution of 320 (V) and 240 (H) pixels. Emissivity was set at 1.0 because it has been reported to induce errors of less than 1°C [20], [21]. All measurements were taken between 11:00 and 13:00 hrs on a single day.

### Pre-processing

**Figure 2:**
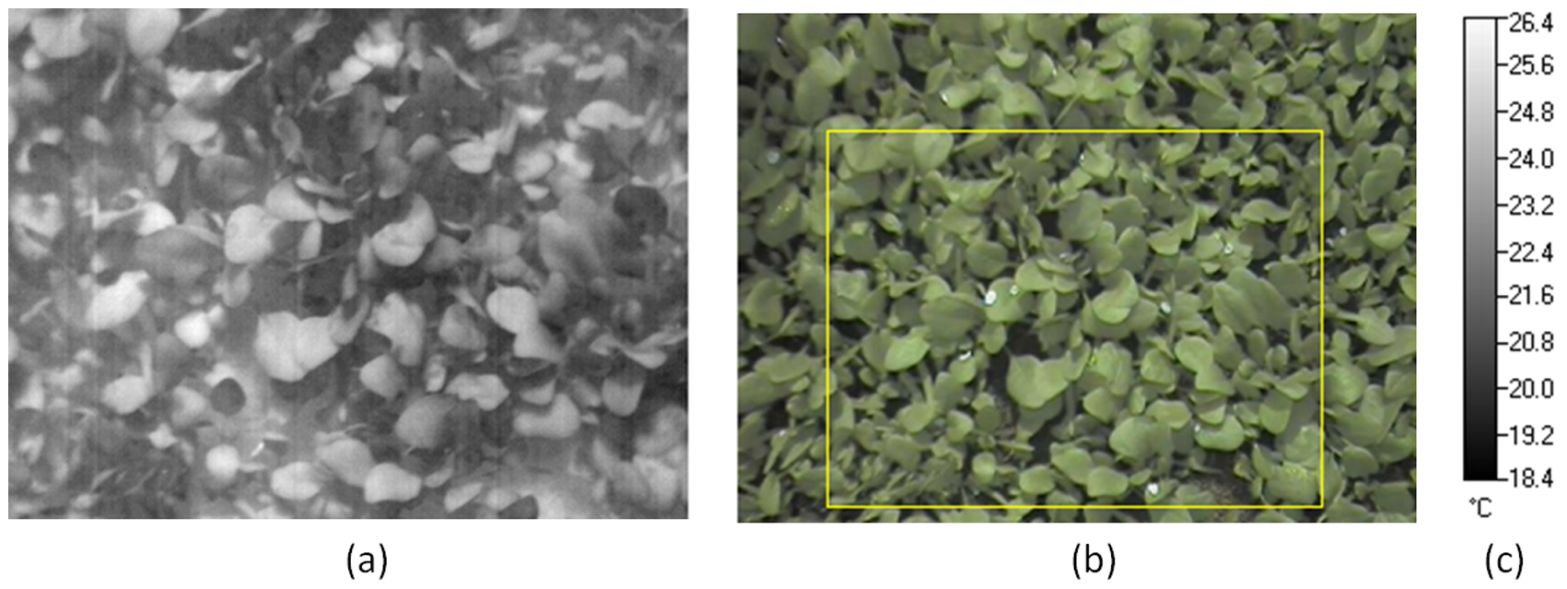
Image(s) obtained using a thermal imaging camera (NEC Thermo TracerTH9100 Pro). (a) thermal image with pixel values ranging from 0–255. (b) Region (rectangle) corresponding to the thermal image in the visible light image. (c) corresponding temperature range.

**Figure 3:**
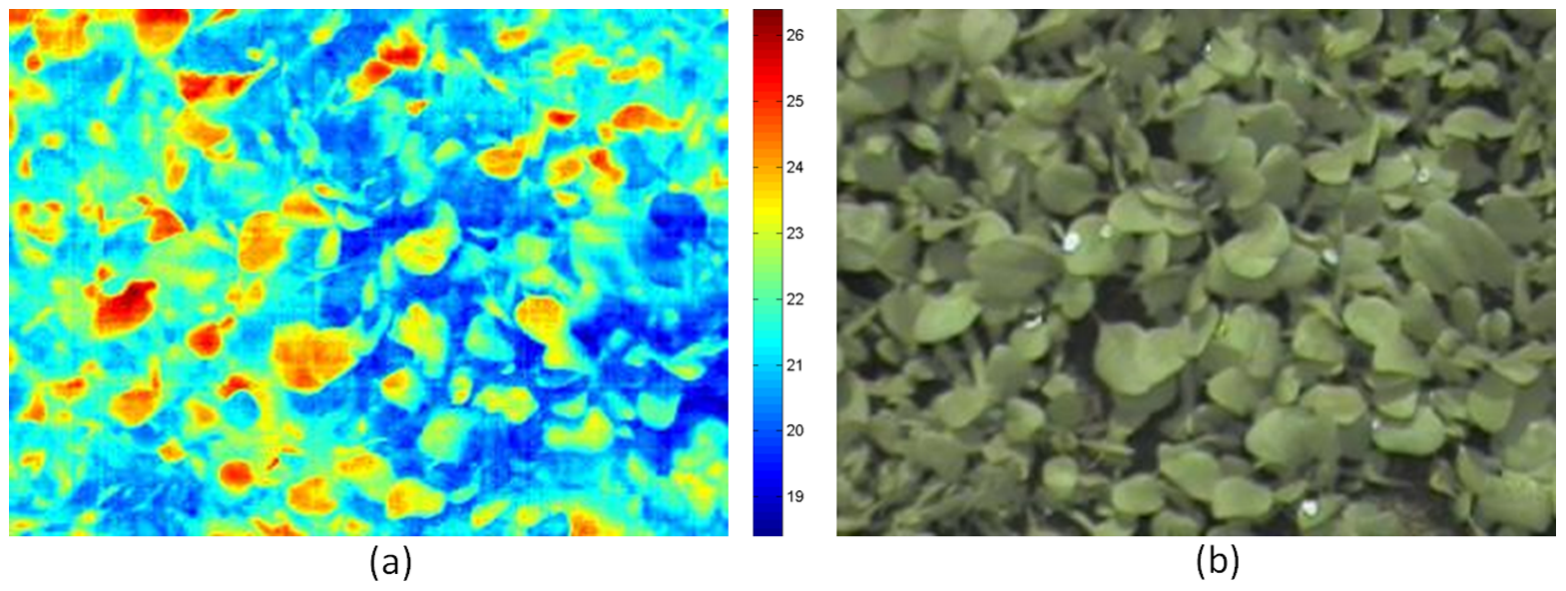
Visible light thermal images of **Figure 2** obtained after pre-processing; (a) the thermal image in **Figure 2**(a) has been replaced by temperature values. (b) visible light image in **Figure 2**(b) has been transformed to match thermal image in a way that same pixel locations correspond to same point located on the plant.

Information from both thermal and visible light images (Figure 2) was used for classification. Thermal images were obtained as images with pixel intensity values ranging from 0 to 255. Initially, the image values were transformed to temperature values. A character recognition algorithm based on cross correlation was used, which automatically recognised the characters in the temperature bar (Figure 2c) and identified the temperature range for the thermal image [22]. This made it possible to replace the image values, which ranged from 0 to 255, with temperature values. In order to extract useful information from thermal and visible light images, both must be aligned so that the pixel location in both images corresponds to the same physical location with respect to the plant. Since both thermal and visible light images are acquired using a single device, there is a fixed transformation between thermal and visible light images. In order to compute this transformation, the transformation between a single pair of thermal and visible light images was calculated by manually selecting control points. To reduce the amount of noise present in the visible light image, anisotropic diffusion filtering was applied [23]. These pre-processing steps resulted in the images shown in Figure 3 and further calculations were conducted on these images.

### Feature Computation

**Figure 4:**
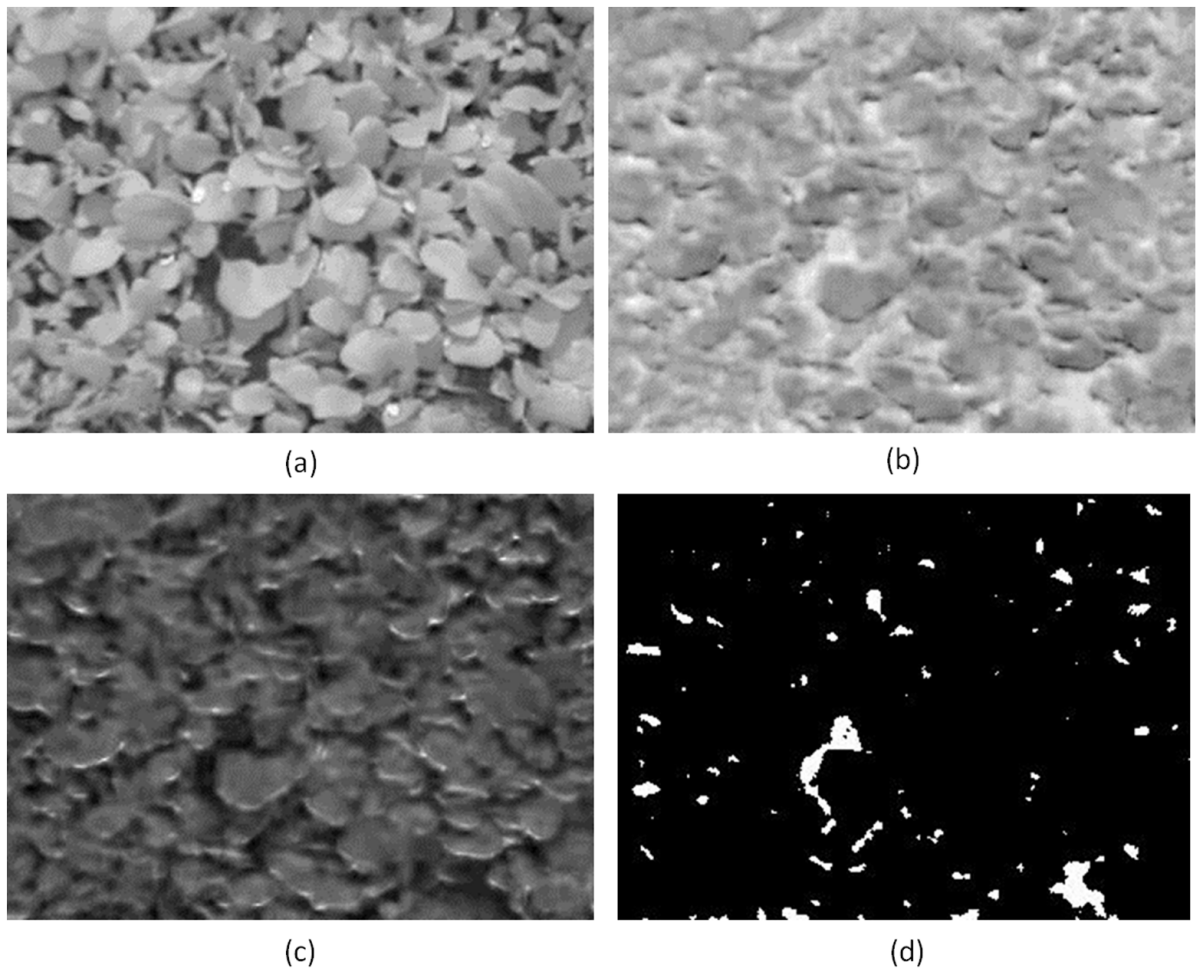
(a), (b) and (c) ‘L’, ‘a’ and ‘b’ channels of the visible light image. (d) thresholded a-channel.

In order to get good classification results, we extracted information from the data in the form of features which carry discriminating information from different treatments and similar information from the same treatment type. Features were selected on the basis of observations made by various researchers [13]–[18]. Average values and variation in the thermal profile of the canopy were selected and combined with information from the visible light image. As a first step, the colour space of the visible light image from RGB to Lab colour space was transformed (Figure 4). In Lab colour space, instead of Red, Green and Blue channels, an L-channel exists for luminance, as well as ‘a’ and ‘b’ channels for the colour components. Features selected for experiments are given in Table 1.

**Table 1:** Features selected for our experiments.

	Symbol	Description	Type	*p*-value
1.	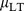	Luminance has been found to be a major factor which affects the thermal profile of an image [16]. In this work the temperature values were linearly scaled (multiply) with the corresponding L-channel of the colour image so that the effect of light intensity was incorporated into the model. After scaling temperature data with the L-channel, mean temperature values of an image was used as a feature.	C/T	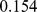
2.	*μ_a_*	The colour information indicates the amount of area covered by the plants or by other types of region. In Figure 4(b), lower intensities corresponded to green parts of the plant whereas the background shows a higher intensity value. For this reason the mean of the a-channel in our set of features was used.	C	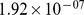
3.	*μ_b_*	Similar to Feature 2, in Figure 4 (c) darker regions corresponded to background and hence the mean of b-channel was included in the set of features.	C	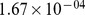
4.	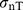	The amount of variation present in an image is also important [30]. Each row of the temperature data was therefore normalised by its median and then the standard deviation of the temperature values employed as a feature, to determine the amount of variation in the canopy region covered by the image.	T	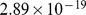
5.	*μ_a_*_T_	In Lab colour space, lower values in a-channel corresponded to green regions. The a-channel was thresholded using Otsu's method [31] to find the background regions as represented by white pixels in Figure 4 (d). Temperature values corresponding to the background were discarded and the mean of the temperature values corresponding to the rest of pixels calculated, as a measure of the mean temperature of green parts of the plant.	C/T	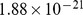
6.	*σ_a_*_T_	Similar to Feature 5, the temperature values corresponding to background were discarded and the standard deviation of temperature values corresponding to the rest of the pixels calculated as a measure of variation in thermal intensities of green parts of the plant.	C/T	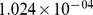
7.	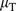	Mean of temperature values	T	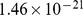
8.	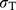	Standard deviation of temperature values	T	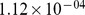

Feature type shows that the corresponding feature contains information about colour (C) or thermal (T) data or both (C/T). The rightmost column shows *p*-values of the features calculated using analysis of variance (ANOVA).

### Support Vector Machines (SVM)

SVM is a supervised learning method used for classification and regression analysis [24]. SVM constructs a hyperplane in high dimensional space and tries to find the hyperplane which maximises the separation between two classes of training data points. In this work, we used linear SVM which uses the model,
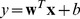
(1)where **x** = [*μ*_LT_,*μ_a_*,*μ_b_*,*σ*_nT_,*μ_a_*_T_,*σ_a_*_T_,*μ*_T_,*σ*_T_] denotes the input feature vector and *y* denotes the classification output (+1 for plants undergoing water stress, and −1 for well-watered plants). SVM models the parameters *b* and **w** to find the maximum margin hyperplane between data points from two classes.

### Gaussian Processes for Classification (GPC)

Gaussian Processes (GP) can be defined as a class of probabilistic models comprised of distributions over functions instead of vectors [25]–[27]. A Gaussian distribution can be expressed by a mean vector and a covariance matrix. A GP is fully characterised by its mean and covariance functions. In machine learning, GPs have been used for regression analysis and classification. Similar to SVM, GPCs also belong to the class of supervised classification methods. However, instead of giving discriminant function values it produces output with probabilistic interpretation, i.e., a prediction for 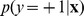 which denotes the probability of assigning a label (*y*) value +1 to the input feature vector **x** [28]. GPCs do not calculate this probability directly on the input variables and assume that the probability of belonging to a class is linked to an underlying GP in the form of a latent function. Given a training set 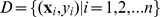 consisting of training images of both classes (water deficit and well-watered), with manually assigned labels *y_i_* to the corresponding feature vectors **x** *_i_* extracted from those images, GPC makes prediction about the label of the feature vector computed from an unseen image 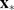, using posterior probability,
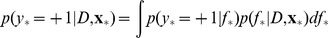
(2)


The probability of belonging to a class 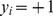 for an input 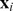 (known data point) is related to the value 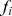 of a latent function *f* [29]. This relationship is defined with the help of a squashing function. In this case, a Gaussian cumulative distribution function was used as the squashing function.
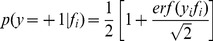
(3)where 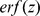 is the error function defined as 
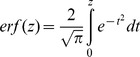
. The second term in the integral in equation (2) is given by,
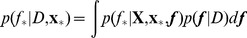
(4)where 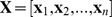 and 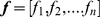, *n* is the number of samples. 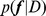 can be formulated by the Bayes' rule as follows,
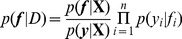
(5)and 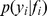 can be calculated by equation (3) and 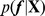 is the GP prior over latent function. Since a GP is characterised by a mean function and a covariance function, a zero mean was used for symmetry reasons, and a linear covariance function selected which has been found to be effective in classification problems [26]. The normalisation term in the denominator is the marginal likelihood given by,
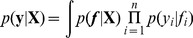
(6)where **y** = {*y*_1_,*y*_2_,….*y*_n_}. The second term in the above equation is not Gaussian and this makes the posterior in equation (5) analytically intractable. However, analytical approximations or Monte Carlo methods can be used. Two commonly used approximation methods are Laplace approximation and Expectation Propagation (EP). EP minimises the local Kullback-Leibler (KL) divergence between the posterior and its approximation and has been found to be more accurate in predicting than Laplace approximation and hence EP was used for approximation in these experiments [25], [26].

## Experiments and Results

### Classification using Machine Learning Methods

**Figure 5:**
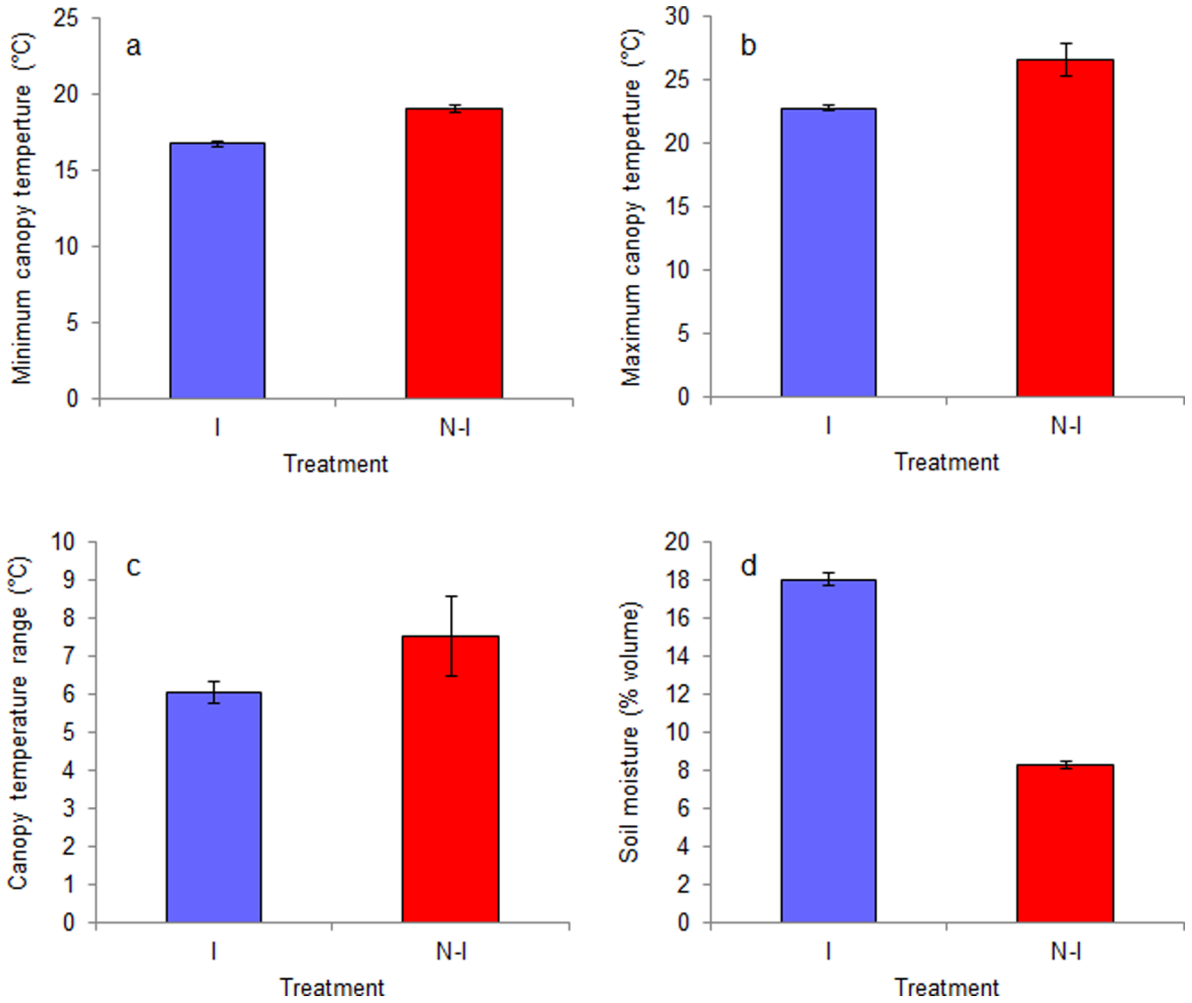
Crop canopy thermal properties (a–c) and soil moisture (d) of irrigated (I) and non-irrigated (N-I) beds of spinach. Crops were grown commercially at Mullens Farm, UK in April 2010. Each bar represents the mean value ± SE n = 3.

A total of 108 images of spinach canopies and corresponding soil moisture point measurements were acquired, with 54 images of well-watered beds and 54 images of droughted beds. The thermal images demonstrated significant variation between the two treatments when judged by soil moisture and thermal canopy properties as taken from the primary thermal images (Figure 5).

Well-watered canopies exhibited lower minimum (F_1,5_ = 59.74, p = 0.002) and maximum (F_1,5_ = 8.71, p<0.05) temperatures than droughted beds. However, the range of temperatures did not differ between treatments when the droughted beds were compared to irrigated spinach plots (F_1,5_ = 1.80, p>0.05). Additionally, it was confirmed that soil moisture differed significantly between treatments (F_1,5_ = 556.19, p<0.0001). All analyses were conducted using 1-way ANOVA.

**Figure 6:**
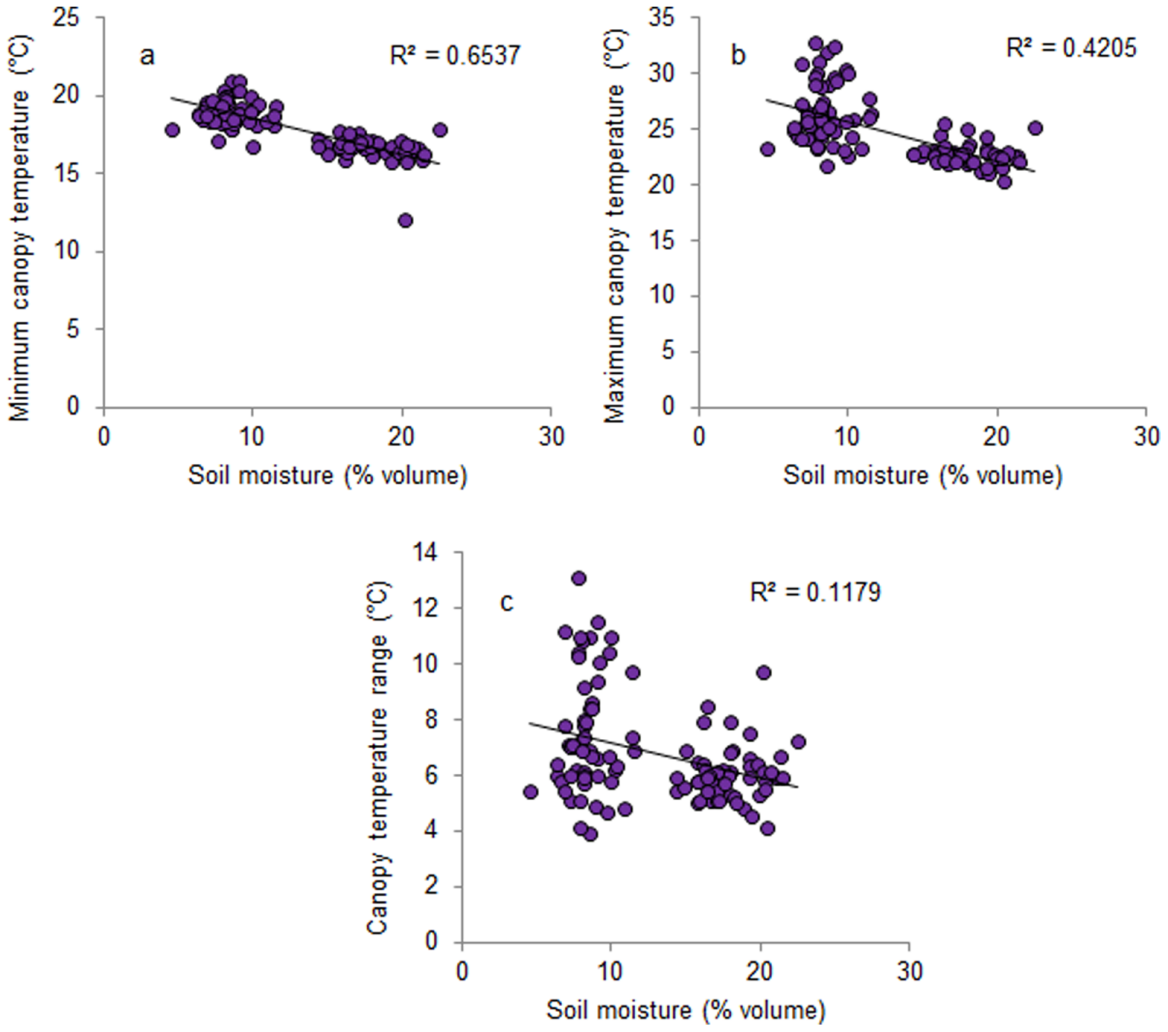
Regressions of crop canopy thermal properties (temperature minimum (a), maximum (b) and range (c)) and soil moisture measurements of irrigated and non-irrigated spinach beds. Crops were grown commercially in April 2010. Trend lines are shown when p<0.005 and the R^2^ value is given.

Regressions demonstrated a number of relationships linking crop canopy thermal properties, taken from the primary thermal images, to direct soil moisture measurements (Figure 6). Moreover, there was a clear segregation into two clusters, accounting for the two treatments. To establish how these relationships interacted, PCA was performed upon the four traits of: soil moisture, minimum temperature, maximum temperature and range of temperature. Components were extracted when their Eigenvalue exceeded a threshold value of 1. One component was extracted which explained 71.6% of total variance (Table 2). This component measured all four traits thus showing their tight coupling and the need for more complex analysis if they are to be used for the detection of soil water deficits. All thermal properties were strongly, positively related to each other while soil moisture was negatively related to all thermal traits. These results implied that the thermal properties of spinach canopies can be used as an indicator of soil water content (Table 3) yet that this approach is not able to accurately detect soil moisture status using primary thermal images. A more complex analysis method is required which is able to utilise both visual and thermal image data to improve soil moisture detection.

**Table 2:** Total variance explained by Principle Component Analysis when both well-watered and droughted spinach crops were measured for their thermal properties (maximum, minimum and range of temperatures) and soil moisture.

Component	Initial Eigenvalues			Extraction Sums of Squared Loadings		
	Total	% of Variance	Cumulative %	Total	% of Variance	Cumulative %
1	2.863	71.567	71.567	2.863	71.567	71.567
2	.830	20.742	92.309			
3	.308	7.691	100.000			
4	6.967E-16	1.742E-14	100.000			

Extraction Method: Principal Component Analysis.

**Table 3:** Component Matrix^a^ from Principle Component Analysis when both irrigated and non-irrigated spinach crops were measured for their thermal properties (maximum, minimum and range of temperatures) and soil moisture.

	Component
	1
Soil moisture	−.750
Minimum temperature	.869
Maximum temperature	.969
Temperature range	.779

**Figure 7:**
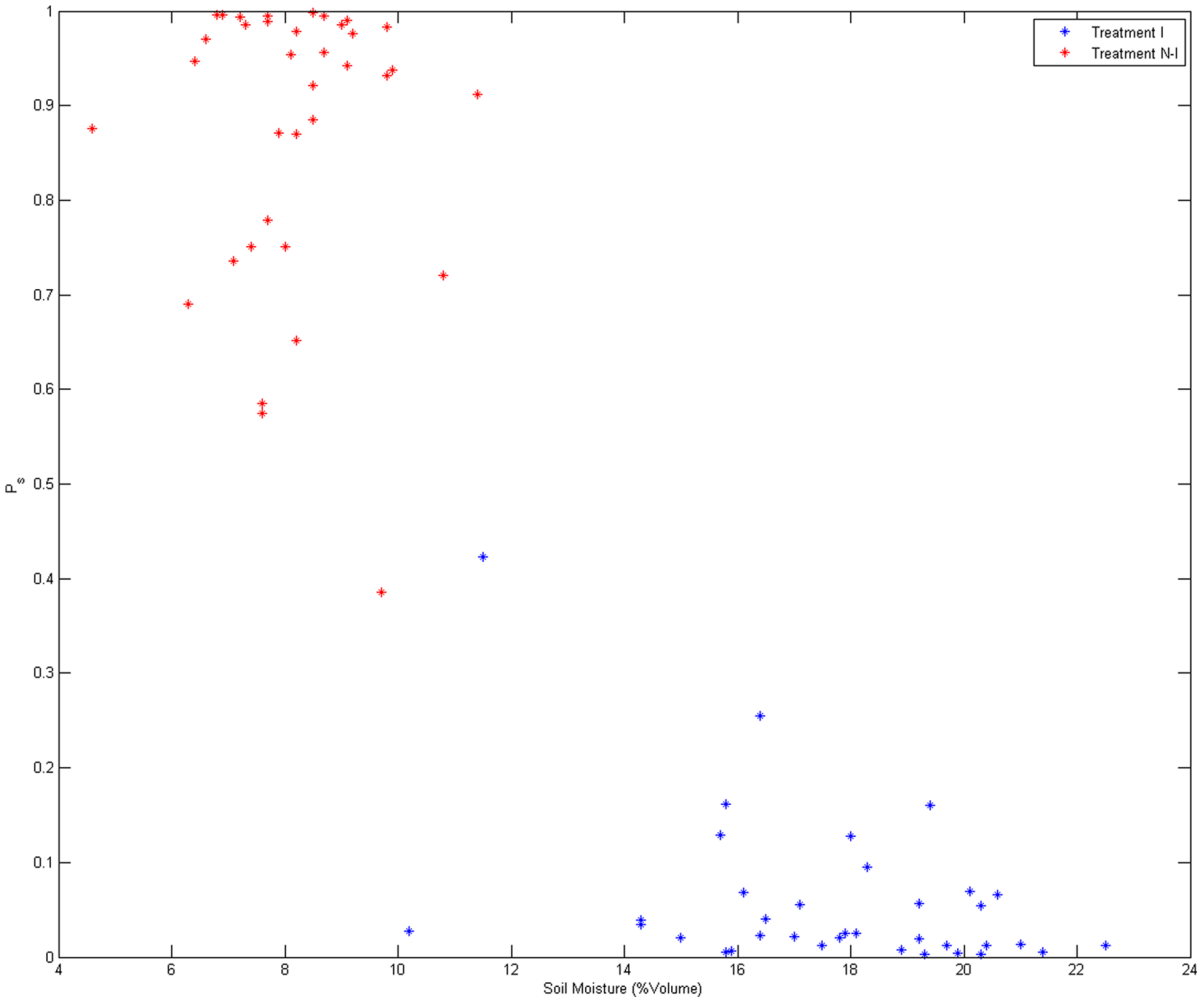
Probability of belonging to treatment N-I (P_s_) versus Soil moisture values (correlation value = −0.89, High moisture means less probability of stress). Soil moisture is given as percentage soil water content v/v. Classification accuracy for this particular set of training and testing data was 98.6% as given by GPC.

The same 108 images of spinach canopies were used for the image processing approach, with the 54 images of well-watered beds being designated treatment I, while the 54 images of the water deficient canopy were designated treatment N-I. The identity of the two treatments was not known during the development of image analysis. After pre-processing, six different features (1–6, Table 1) were obtained from each image. SVM and GPCs were used to classify the test images into water deficient and well-watered. For SVM linear kernel was used and for GPC a zero mean and a linear covariance function were chosen. As discussed before, SVM gives discrete classification results and classifies each image as treatment I or treatment N-I, whereas GPC gives the probability (likelihood) of each image belonging to a particular treatment. Figure 7 shows the probability of an image belonging to treatment N-I (P_s_) versus the values of soil moisture for one set of training and testing data. It was clear that the probability (P_s_) was highly related to manually calculated soil moisture values (correlation value = −0.89 for Figure 7). Based on the probabilities given by GPC, each image was classified as an image from either treatment I or treatment N-I.

Since two different types of classifier were used, disagreement between the results of both the different classifiers could be assessed, which occurred in some cases. This disparity was utilised to further refine the classification results; although this refinement is not very significant, it produces better results. Information from both classification methods was combined to reduce the error from classification. If an image was classified by SVM as treatment I and its probability of belonging to treatment N-I according to GPC was higher than 80% then this image was classified as treatment N-I. On the other hand, if an image was classified as treatment N-I and its probability according to GPC was less than 20%, the image was classified as treatment I. It was found experimentally that the 80–20% threshold gave the best results.

200 iterations were employed to test the accuracy of the classifiers for different pairs of training and testing sets. In each iteration, 36 images were chosen at random (18 from each treatment) for training purposes and the proposed algorithm was tested on the other 72 images. Results showed that GPC demonstrated a higher level of accuracy than the SVM classifier (Table 4); however if information from the results of both of the classifiers was combined, results were improved in terms of sensitivity, specificity, positive predictive value (PPV) and accuracy. An average accuracy of 96.3% was obtained for SVM, 96.7% by using GPC and a slightly higher 97.1% when information from both classifiers was combined. When the results of colour-only and temperature-only features were compared, it was found that combining information from both temperature and colour data increased the accuracy of classification. Furthermore, including mean and standard deviation of temperature values without combining them with colour information diminished the accuracy of results; thus the mean (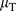) and standard deviation (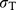) were removed from the set of features.

**Table 4:** Comparison of average classification results of different classifiers using the proposed set of features.

Feature(s) selected	Classifier	Sensitivity (%)	Specificity (%)	PPV (%)	Accuracy (%)	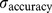
Color only (*μ_a_*, *μ_b_*)	SVM	67.28	70.29	70.98	67.74	3.36
	GPC	80.68	52.96	21.68	56.87	3.55
	Both Classifiers	67.32	70.42	71.11	67.80	3.40
Thermal only ( 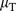 , 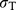 )	SVM	93.35	91.28	90.89	92.14	1.92
	GPC	93.06	80.30	76.67	85.42	2.29
	Both Classifiers	93.35	91.28	90.88	92.14	1.92
Features (1–8) Table 1.	SVM	95.52	96.39	96.30	95.85	1.97
	GPC	96.38	97.39	97.30	96.79	1.56
	Both Classifiers	96.62	96.93	96.84	96.70	1.60
Features (1–6) Table 1.	SVM	95.86	96.86	96.80	96.27	1.58
	GPC	96.53	96.99	96.90	96.68	2.00
	Both Classifiers	**96.97**	**97.38**	**97.31**	**97.12**	**1.52**

**Figure 8:**
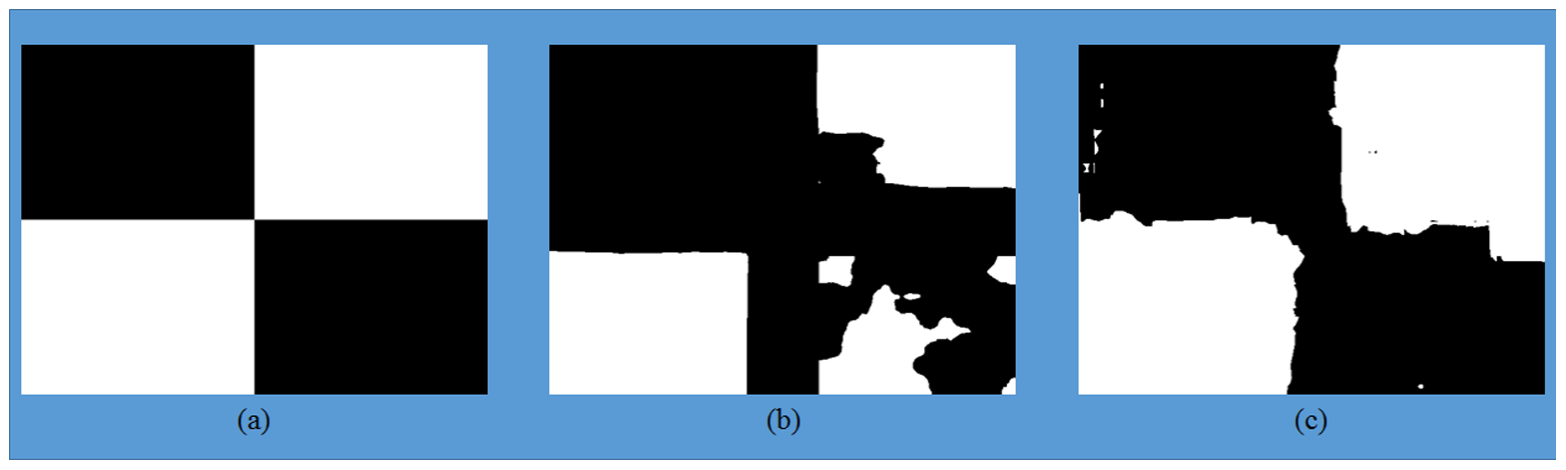
(a) The ground truth pattern for mixed condition mosaicked image. Black colour represents image region corresponding to treatment I and white colour represents the image region which corresponds to treatment N-I. (b) & (c) show classification results obtained using combined classifier with thermal only and proposed feature set respectively.

**Figure 9:**
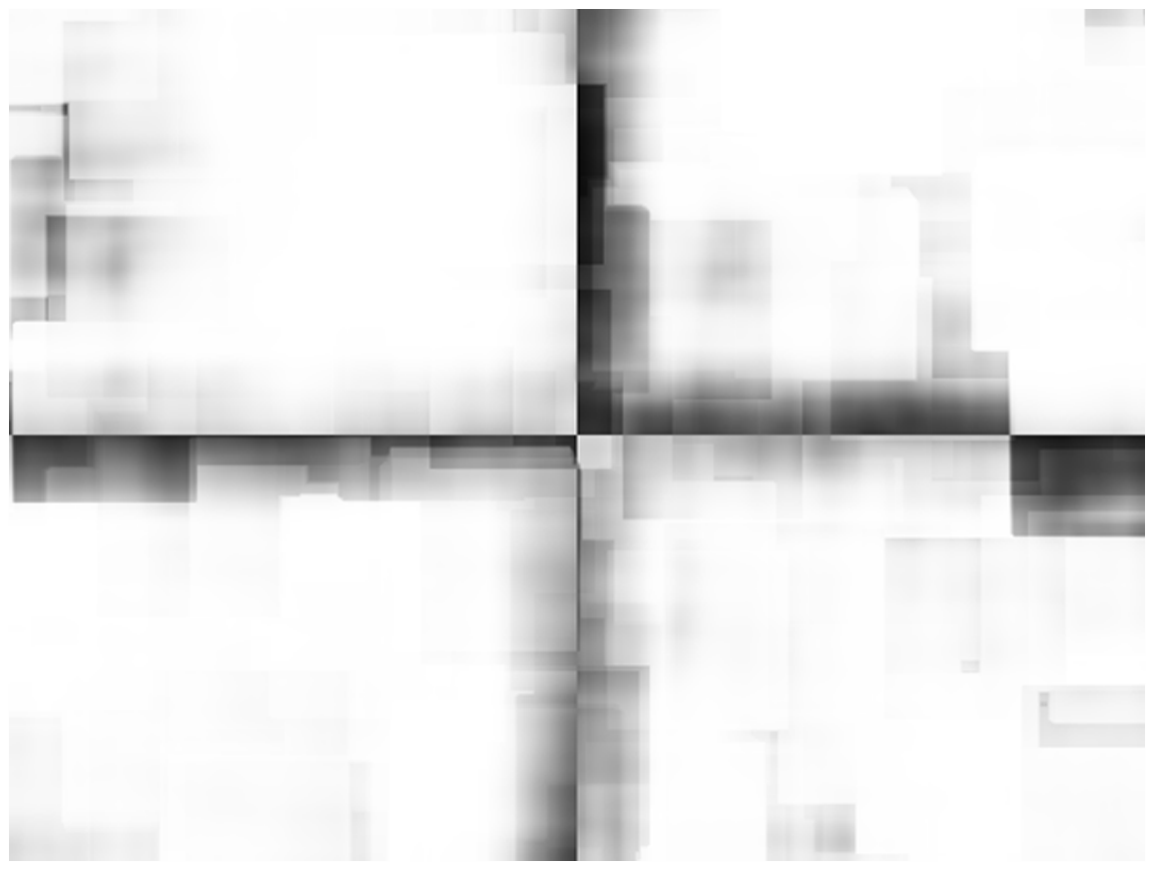
GPC classification result in terms of confidence score (C_s_). Bright shade represents high confidence in classification results and dark shade represents low confidence in the classification. The classifier has higher confidence in the region with image from treatment I or treatment N-I, however the confidence value is low, as depicted by darker shade, around the boundary of two merging images from different treatments.

To further investigate the strength of classifier with the proposed set of features, we created an artificial image with mixed conditions by combining randomly picked thermal and visible light images from Treatment I and Treatment N-I to form a mosaic. The ground truth pattern for the mosaicked image is shown in **Figure 8** (a). Black colour represents image region corresponding to treatment I and white colour represents the image region which corresponds to treatment N-I. A block of size 50×50 pixels was defined at each pixel location in the mosaicked image and the classifier was tested using the features extracted from each of these small blocks (307,200 blocks in total). The classifier for this experiment was trained in a similar way as for the real data (i.e., on randomly selected 36 original images). By using 50×50 blocks to simulate mixed conditions, we reduced the amount of information available, so the accuracy of classification is expected to deteriorate. However, the results show robustness of our proposed feature set when compared to thermal only features. The classification results using the combined classifier with thermal only and the proposed feature set are shown in **Figure 8** (b) & (c) respectively. The classification accuracy using SVM, GPC and the combined classifier was calculated to be **89.1%**, **94.1%** and **92.5%** using the proposed feature set compared to **78.3%**, **54.1%** and **76.3%** when using thermal only features. The classification accuracy for the combined classifier is less than GPC in the proposed feature set and less than SVM in the thermal only feature set in mixed conditions, however, we still consider this classifier to be important as it gives the best results on real data. **Figure 9** shows GPC classification results using the proposed set of features in terms of the confidence score (C_s_). For treatment I, C_s_ = 1 – P_s_ and for treatment N-I, C_s_ = P_s_, where P_s_ is the probability of belonging to treatment N-I as given by GPC. The bright shade represents high confidence in classification results and dark shade represents low confidence in the classification. It can be observed that the classifier has higher confidence in the region where the image is from treatment I or treatment N-I, however the confidence value is low, as depicted by low grey values around the boundary of two merging images from different treatments. The mean and standard deviation of C_s_ was calculated to be **90.5%** and **17.8%** using proposed feature set and **51.1%** and **32.3%** using thermal only features respectively.

## Discussion and Conclusions

Our results show that by combining information from thermal and visible light images and using machine learning techniques, canopies which are experiencing water deficits can be identified with high accuracy – more than 97%. Thus we have considerably improved the use of remote images in the detection on canopy stress using this combined approach. The purpose of this study was to test a new dimension of automated classification methods for the detection of regions of a crop canopy that are responding to soil water deficit and to go beyond the restrictions of commonly used statistical approaches. We showed that extraction of a good set of image features can be useful for classifications of this type. In this study, we were able to detect regions of the canopy which were experiencing soil moisture deficit by using a machine learning approach instead of stress indices. Initially, the effect of reflected light and background information was reduced in order to extract features. In the second step these features were classified using SVM, GPC and a combination of both classifiers. The colour information in visible light images provides information about the amount of reflected light intensity from the plant. Using this information, temperature values were scaled on the basis of reflected light. Plant regions can also be identified in the registered thermal image using colour information. This helped to discard temperature values belonging to the background and extract useful information from plant regions in [15]. Based on information from visible light and thermal images, a worthy set of features can be extracted. In these experiments, it was found that scaling with luminance intensity (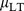) plays an important role in classification. When the luminance intensity scaling feature was removed from our set of features, we found that the accuracy of the classifiers decreased (Table 5). In the case of GPC classification, accuracy fell by up to 7%. This showed that the selection of suitable features is critical when data from thermal images are classified for stress analysis. We have also tested the proposed classifier on an artificially generated mixed condition image. The classification results in this image show a significant improvement using the proposed feature set when compared to the thermal only feature set. We found the proposed set of features robust to amount of input information and to mixed-condition images.

**Table 5:** Comparison of average classification results of different classifiers without using light intensity scaling feature (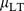).

	Sensitivity (%)	Specificity (%)	PPV (%)	Accuracy (%)	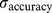
SVM	94.98	95.01	94.83	94.84	2.01
GPC	88.21	91.84	92.05	89.70	2.61
Both Classifiers	95.28	95.27	95.08	95.12	1.89

In the future, we plan to extend this work to identify canopies under multiple levels of stress. Furthermore, information about leaf angles and distance of the plant from the camera will be used to estimate a more accurate model of the thermal profile, which in this case was linear scaling with light intensity values. For information about depth and leaf angles, a stereo image setup is needed in order to model the effect of leaf angles and distance of leaves from the camera. This model can be combined with more sophisticated machine learning techniques for early water stress detection in crops, and, if automated, could be used to improve irrigation efficiency by optimising the timing and spatial distribution of irrigation events. Other plant stresses such as disease could also potentially be detected rapidly and pre-symptomatically using these methods.
